# Co-subsistence of avian influenza virus subtypes of low and high pathogenicity in Bangladesh: Challenges for diagnosis, risk assessment and control

**DOI:** 10.1038/s41598-019-44220-4

**Published:** 2019-06-05

**Authors:** Rokshana Parvin, Jahan Ara Begum, Emadadul Haque Chowdhury, Mohammed Rafiqul Islam, Martin Beer, Timm Harder

**Affiliations:** 1 Federal Research Institute for Animal Health, Friedrich-Loeffler-Institut (FLI), Suedufer 10, 17493 Greifswald-Insel Riems, Germany; 2 Department of Pathology, Faculty of Veterinary Science, Bangladesh Agricultural University, Mymensingh 2202, Mymensingh, Bangladesh

**Keywords:** Influenza virus, Pathogens

## Abstract

Endemic co-circulation of potentially zoonotic avian influenza viruses (AIV) of subtypes H5N1 and H9N2 (G1 lineage) in poultry in Bangladesh accelerated diversifying evolution. Two clinical samples from poultry obtained in 2016 yielded five different subtypes (highly pathogenic [HP] H5N1, HP H5N2, HP H7N1, HP H7N2, H9N2) and eight genotypes of AIV by plaque purification. H5 sequences grouped with clade 2.3.2.1a viruses while N1 was related to an older, preceding clade, 2.2.2. The internal genome segments of the plaque-purified viruses originated from clade 2.2.2 of H5N1 or from G1/H9N2 viruses. H9 and N2 segments clustered with contemporary H9N2 strains. In addition, HP H7 sequences were detected for the first time in samples and linked to Pakistani HP H7N3 viruses of 2003. The unexpected findings of mixtures of reassorted HP H5N1 and G1-like H9N2 viruses, which carry genome segments of older clades in association with the detection of HP H7 HA segments calls for confirmation of these results by targeted surveillance in the area of origin of the investigated samples. Hidden niches and obscured transmission pathways may exist that retain or re-introduce genome segments of older viruses or reassortants thereof which causes additional challenges for diagnosis, risk assessment and disease control.

## Introduction

Avian influenza viruses (AIVs) remain important pathogens for wild birds, domestic poultry and humans. AIVs group into 16 hemagglutinin (HA) and 9 neuraminidase (NA) subtypes each revealing various lineages and host-specific adaptations^1^. Based on the pathogenic potential in experimentally infected chickens, phenotypes of low and high pathogenicity are distinguished (LP/HPAIV). Several AIV subtypes and lineages exhibiting explicit zoonotic propensity have emerged as a public health threat^2^. Notably, these are goose/Guangdong (gs/GD) lineage derived HPAI H5 viruses and 2013-origin Chinese LPAI H7N9 viruses, but also further viruses such as the G1 lineage of subtype H9N2 have caused sporadic human cases^3,4^. Within the last decade, gs/GD HPAI H5 and LPAI H9N2 viruses have become endemic in poultry in several Asian and African countries causing huge economic losses^4,5^. Domestic duck populations play an important role in establishing and maintaining endemicity as they often subclinically shed and spread these viruses^1,6,7^. Long term co-circulation of multiple influenza virus lineages in different species of poultry and inconsequent control attempts including fragmentary vaccination coverage has facilitated diversifying evolution by genetic drift and reassortment^8–10^.

Bangladesh, a south Asian country with a high density of poultry and human populations is no exception in this respect. Potentially zoonotic HPAI H5N1 and H9N2 viruses co-circulate endemically in commercial and backyard poultry shuttling between different poultry species and causing sporadic spill-overs into wild bird populations since early 2007^11–14^. Several reassortants between HPAI H5N1, H9N2 and HPAI H7N3 viruses have been recorded in the country although some reassortments took place before incursion into Bangladesh^11–13^. In addition, Bangladesh suffered from successive incursions and replacements of three different gs/GD HPAIV H5N1 clades, 2.2.2, 2.3.2.1, and 2.3.4.2^14^. Since 2012, clade 2.3.2.1a viruses have replaced previously co-circulating gs/GD viruses^15,16^. Exposure of humans to these viruses is emphasized by nine and three cases of human infection with HPAIV H5N1 (including one death) and H9N2, respectively, till to date^17–19^.

Here we illustrate the intricate epidemiological situation and the evolutionary potential of co-circulating AIV subtypes of different sub-, geno- and phenotypes by molecular analyses of clinically overt AI outbreaks in commercial and backyard poultry in Tangail district, one of the most crowded districts for both poultry and human populations at the northern perimeter of Dhaka, the capital of Bangladesh.

## Results

### Clinical and pathological observation

**Figure 1:**
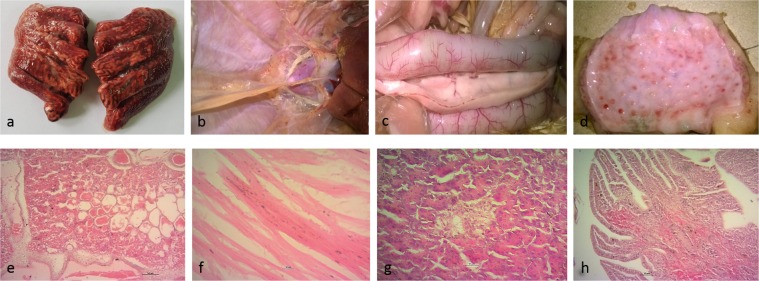
Gross and microscopic lesions in chicken, upper row, (**a**) congested lung; (**b**) petechial and ecchymotic haemorrhages on the air sac and epicardium; (**c**) pancreas with dark red areas along its length and congested blood vessels at the wall of the intestine; (**d**) mucosal haemorrhages in proventriculus; lower row, (**e**) severe congestion and edema in lung tissue; (**f**) mild cardiac myodegeneration; (**g**) necrosis of pancreatic islets; (**h**) haemorrhages in duodenal mucosa.

Limited HPAI syndrome surveillance was carried out in Tangail district throughout January to May 2016 in commercial medium size layer chicken farms (so-called sector 3 according to the Food and Agriculture Organization of the United Nations [FAO]) reporting respiratory illness. Carcasses of four chickens (VP02-VP05) were sampled at four different farms keeping grower and layer chickens 6 to 84 weeks old, cage-reared and putatively vaccinated against H5 viruses (unknown source). From neighboring backyard poultry holding the carcass of a duck (VP06) was collected as well. The sampled sector 3 chicken layer farms, each of up to 2000 birds reported morbidity of up to 90% dominated by respiratory disease and reduced egg production. Cumulative mortality approaching 35% at these farms was substantial but still low compared to previously recorded HPAI outbreaks in chickens in Bangladesh^20^. Clinical presentation and epidemiological considerations thus raised the suspicion of AIV H9N2 infections. This suspicion was corroborated by contemporary, laboratory-confirmed findings of H9N2 cases with similar clinical presentation in the same area (unpublished). This prompted submission of samples directly to FVS-BAU while samples from HPAI suspect cases are to be filed to the National Reference Laboratory at Bangladesh Livestock Research Institute. At necropsy, however, at FVS-BAU, severe congestion in lung and trachea along with severely congested blood vessels at intestinal walls, petechial to ecchymotic haemorrhages at the epicardium, air sacs, intestine, proventricular glands and pancreas dominated (Fig. 1a–d). Histopathologically, widespread haemorrhages, congestion and massive edema in lungs, mild cardiac myo-degeneration, hemorrhages in the lamina propria of intestine and necrosis of pancreatic islets were recorded (Fig. 1e–h) and evoked the impression of a systemic infection. AIV RNA was detected by M-specific conventional RT-PCR while samples were negative for Newcastle disease virus (NDV).

Additional samples, came from a commercial chicken farm (C1/2016) and a backyard duck (D1/2016) sampled in November 2016 in Mymensingh district bordering Tangail. Similar clinical signs and lesions as described above were recorded in the respective chickens and ducks (data not shown). However, mortality in the commercial layer chicken farm of C1/2016 was higher (70–75%). In this farm, in contrast to the flocks in Tangail district, an HPAIV H5N1 mono-infection was diagnosed. Mortality in Tangail farms was lower (35%) and, initially, an H9N2 infection was diagnosed that later turned out to be a mixed infection comprising HPAIV H5 and H7.

### Viral sequence and phylogenetic analyses directly from clinical samples

**Figure 2:**
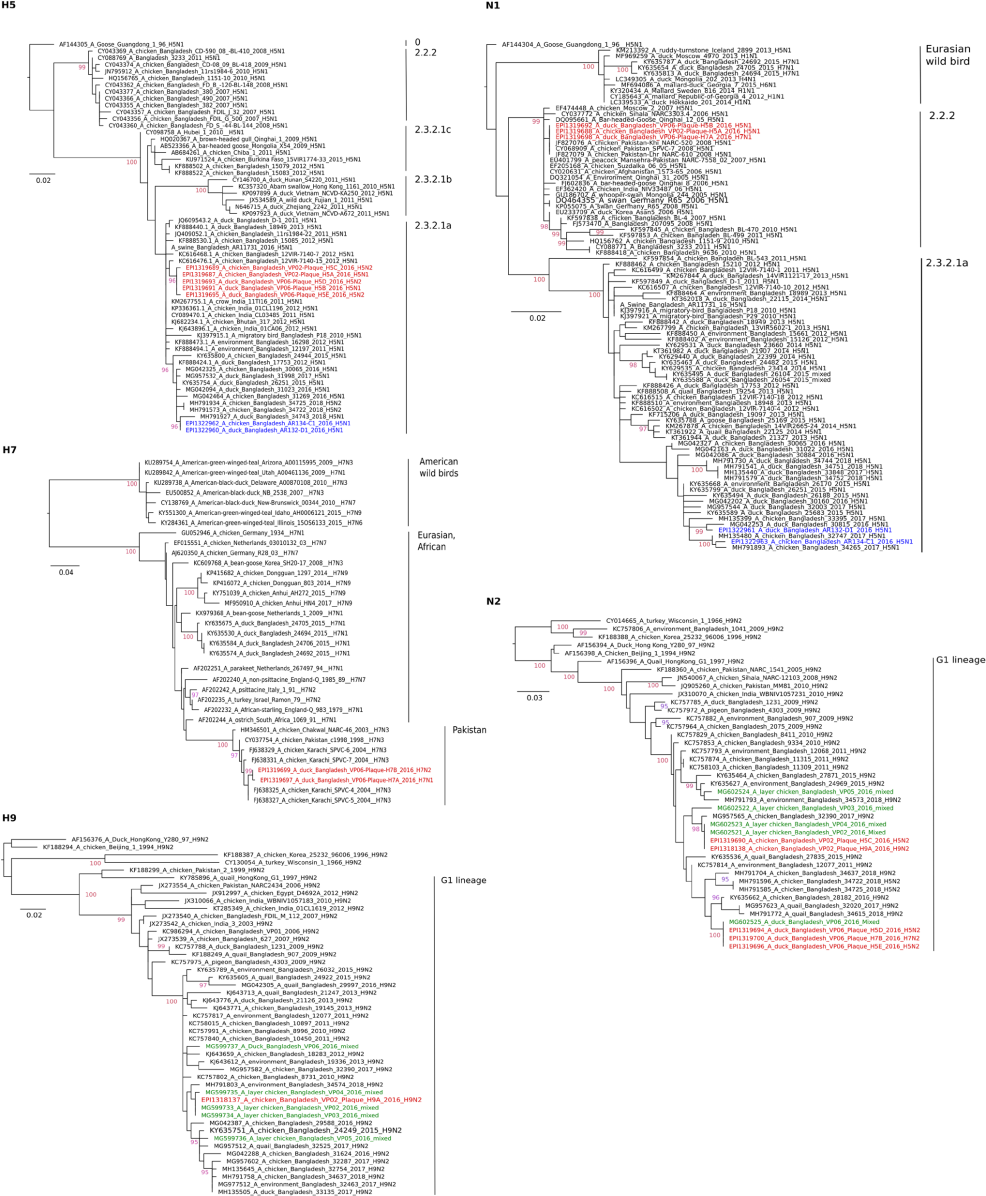
Phylogenetic trees of HA H5, H7, H9, and NA N2 and N1 of sequences established from clinical samples (green) and plaque-purified viruses (red) from Tangail district. Sequences of samples from Mymensingh district are shown in blue. Maximum likelihood trees were established using the IQ-Tree software. Amino acid alignments of the HA1 and the full length NA proteins were used for calculation based on the specific FLU substitution model recommended by Dang *et al*.^44^. Robustness of branching orders was tested by an ultrafast bootstrap approximation algorithm^38^. Trees are drawn to scale and clading information is shown to the right of each tree.

**Figure 3:**
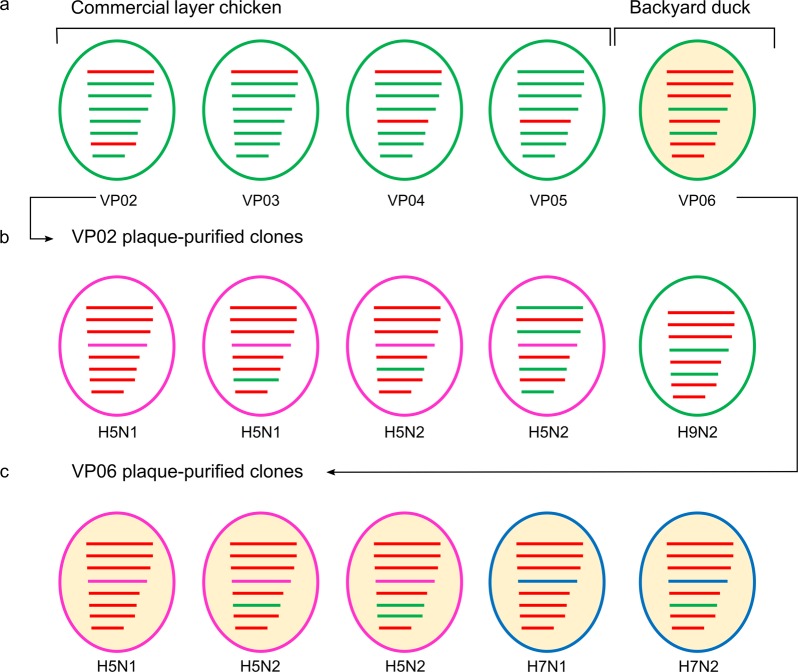
Sub- and genotype characterization of avian influenza viruses detected in original clinical samples (**a**) and following virus isolation and plaque purification (**b**,**c**) from selected chicken and duck origin samples, respectively. Genetic relationship/origin of segments is color-coded, G1/H9N2 - green, 2.2.2/H5N1 - red, 2.3.2.1a/H5N1 – pink, Pak/H7N3- blue.

**Figure 4:**
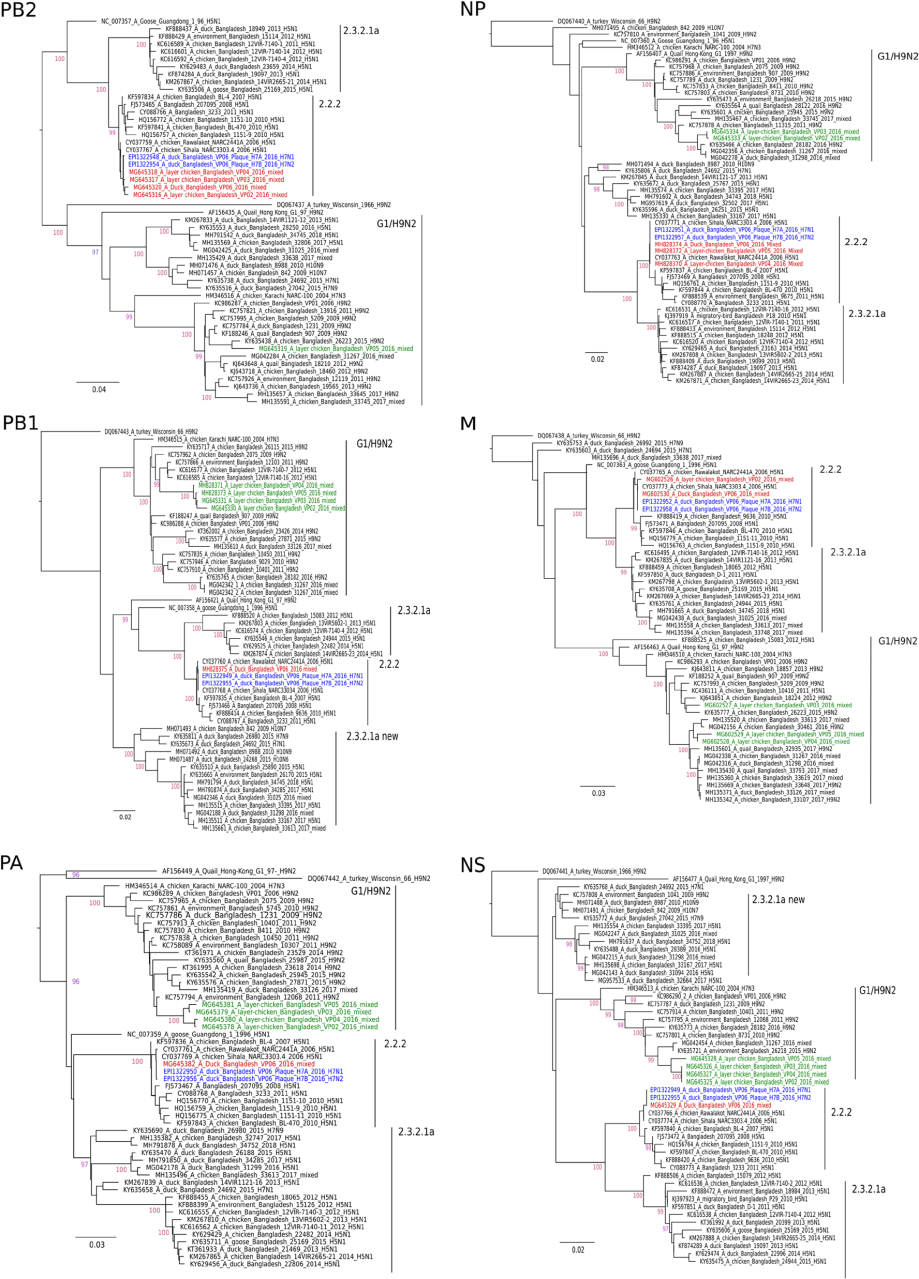
Phylogenetic trees of PB2, PB1, PA, NP, M and NS gene segment sequences established from clinical poultry samples from Tangail district. Maximum likelihood trees were established using the IQ-Tree software. Nucleotide sequence alignments of the respective internal genome segments were used for choosing the best-fitted model according to Bayesian information criterion analysed by ModelFinder (embedded in IQ-Tree)^45^. Robustness of branching orders was tested by an ultrafast bootstrap approximation algorithm^38^. Trees are drawn to scale and clading information is shown to the right of each tree. G1/H9N2-like segments are depicted in green and gs/Gd 2.2.2-like segments are in red color. Sequences of the H7N1 and H7N2 plaque-purified viruses are presented in blue.

At FVS-BAU, all eight AIV genome segments were successfully amplified and full-length genome sequences obtained from clinical samples of four layer chicken farms (VP02-VP05) and the backyard duck (VP06) from Tangail district. Sequence analysis suggested presence of H9N2 viruses with a dibasic HA cleavage site motif (KSKR/GLF) in all samples. HA H9 and NA N2 phylogenetic analyses revealed that all sequences were similar to circulating Bangladeshi H9N2 viruses of the G1 lineage (Fig. 2, H9, N2, green color). Sequences obtained from chicken samples but not the duck sample revealed the Q234L substitution (H9 numbering) known to extend HA binding to alpha 2,6 sialic acid ligands^21,22^ (Supplementary Data; Table S1). Depending on the sample at least one or more of the internal genes appeared to derive from reassortment with HPAIV H5N1, clade 2.2.2 (Figs 3a, 4 & Supplementary Data; Table S2). Samples C1/2016 and D1/2016 from Mymensingh district were identified as contemporary gs/GD H5N1 viruses of clade 2.3.2.1a (Fig. 2, H5, N1, blue color).

### Genotypic and phenotypic characterization of co-isolated AI viruses

Direct Sanger sequencing of clinical samples (VP02-VP06) suggested presence of reassorted H9N2 viruses. In contrast, full range RT-qPCR-based subtyping by the “Riems influenza A typing assay” (RITA) carried out on RNA extracted from allantoic fluid of embryonated chicken egg (ECE) passage detected further AIV HA and NA subtypes H5, H7, H9, N1 or N2 for samples VP02-06 from Tangail district. No further co-infection was detected for H5N1 positive samples C1/2016 and D1/2016 obtained from Mymensingh district (Table 1). Virus isolation and plaque purification flanked by RITA was used exemplarily for samples ck/VP02 and dk/VP06 to confirm co-mingling of several subtypes in the same sample. To avoid cross contamination, samples were treated separately on different days.

**Table 1:** Co-infection with up to three HA subtypes detected in poultry samples from Tangail but not from Mymensingh district, Bangladesh, by use of arrayed subtype-specific RT-qPCRs (RITA, 23).

Sample	Origin	M	H5	H7	H9	N1	N2
A/layer chicken/BD/VP02/2016	Tangail	13.07	19.4	27.1	14.24	20.46	14.35
A/layer chicken/BD/VP03/2016	Tangail	16.38	17.45	29.82	19.03	18.19	19.26
A/layer chicken/BD/VP04/2016	Tangail	16.34	19.71	Neg	17.17	19.72	18.39
A/layer chicken/BD/VP05/2016	Tangail	30.25	31.86	Neg	34.54	32.3	32.32
A/duck/BD/VP06/2016	Tangail	15.57	17.69	18.91	19.86	17.55	17.61
A/chicken/BD/C1/2016	Mymensingh	20.23	13.24	Neg	Neg	14.04	Neg
A/duck/BD/D1/2016	Mymensingh	18.02	16.82	Neg	Neg	17.28	Neg

The results are presented as RT-qPCR Cq values; Neg – Cq > 40.

From a total of 300 plaques obtained at three consecutive passages without amplification between each plaque round, ck/VP02 yielded pure plaques of subtypes H5N1, H5N2, and H9N2 of five different genotypes (Fig. 3b). Four subtypes (H5N1, H5N2, H7N1, H7N2) and five different genotypes were rescued from the backyard duck isolate dk/VP06 (Fig. 3c).

H5 HA genes of plaque-purified H5N1 or H5N2 (Purified from VP02 and VP06 sample originating from Tangail district) clustered with HPAIV clade 2.3.2.1a (HA cleavage site PQRERRRKR/GLF) maintaining a close relationship with H5N1 viruses described in Bangladesh during 2012–2013 but distinct from recently circulating H5N1 sample C1/2016 and D1/2016 originating from Mymensingh district in late 2016 (Fig. 2, H5). The HA gene of purified H7 viruses (H7N1 or H7N2) of the duck sample (VP06) surprisingly clustered with HPAIV isolated during the early 2000s in Pakistan (Pak/H7N3) (Fig. 2, H7). The presence of a polybasic HA cleavage site motif PETPKRRKR/GLF confirmed the HP character of plaque-purified H7 clones. The H9 HA gene of plaque-purified H9N2 viruses was very similar to the ones generated directly from the clinical samples (Fig. 2, H9).

The NA N1 segment in both H5N1 and H7N1 plaques was closely related to H5N1 viruses from Bangladesh and Pakistan which were detected during 2006–2008 and belonged to gs/GD clade 2.2.2 (Fig. 2, N1), clearly distinct from the N1 of clade 2.3.2.1a. NA N2 in H5N2, H7N2 or H9N2 derived from circulating G1/H9N2 viruses (Fig. 2, N2).

The genotype of the plaque-purified viruses was determined by sequencing-independent SYBR-Green RT-qPCRs assigning the internal gene segments to either the G1/H9N2 or 2.2.2/H5N1 origin (Fig. 3b,c). None of the internal genes of the clinical samples (VP02-VP06) and of the plaque-purified viruses was found similar to the currently circulating 2.3.2.1a/H5N1.

The rescued reassortant subtypes exhibited distinct plaque morphology. The H5N1 subtype produced the largest plaques at approximately 4.5 mm in diameter. Plaque area measurements using ImageJ^23^ revealed smaller plaques compared to H5N1 for H7N1 (55%), H9N2 (16%), H5N2 and H7N2 (1.0–1.8%). HP H5N1, H5N2, H7N1 and H7N2 produced higher virus yields (TCID_50_ of 10^7^ to 10^9^ per mL allantoic fluid) and a shortened mean-death time (MDT, 48 hours post inoculation) compared to purified H9N2 (TCID_50_ of 10^6^ per mL; MDT 80 hours post inoculation).

### Approximation of viral fitness using an *in ovo* competition model

Given the co-presence of up to three different AIV subtypes of LP and HP phenotypes with a seemingly dominance of G1/H9N2 in the clinical samples, the relative fitness of different sub- and genotypes was examined in ECE co-infected with up to three distinct sub- or genotypes at the same TCID_50_ of 10^3^/0.2 mL. Independent of the sub- and genotype none of the co-inoculated clones was outcompeted during three consecutive blind passages. In particular, seemingly unconstrained growth of H9N2 clones was noted even in the presence of H5 or H7 HPAI viruses. In competitions between HP H5N1 and HP H5N2, the N2 gene became dominant, i.e. Cq-values for N1 and N2 were similar in the inoculum at passage 0 but differed by 7–9 Cq values at passage 3 with N2 taking advantage over N1 (Supplementary Data; Table S3). Also in another competition between HPAIV H5N1 and H9N2, N2 fully superseded N1. In triple co-infection experiments (H5N1, H7N1 and H9N2) equal amplification of the three HA and the two NA segments were evident over three passages (Supplementary Data; Table S3). The origin of internal gene segments (H9N2 versus H5 2.2.2) did not reveal clear advantages of certain segments.

## Discussion

In total, five subtypes comprising eight different genotypes were discernable by RT-qPCR subtyping (RITA), virus isolation and after plaque purification from two selected clinical poultry samples (ck/VP02 and dk/VP06) of AI outbreaks. The same samples were previously diagnosed based on RT-PCR and direct Sanger sequencing of clinical material, to be due to infection with reassorted H9N2 viruses. Despite presence of gene segments of several sub- and genotypes in the same sample, the corresponding sequence chromatograms showed a high quality; this is probably due to the high subtype specificity of primers used for cycle sequencing which grossly favored a single subtype over the rest. H5N1, H5N2 and H9N2 viruses were purified from ck/VP02 whereas H5N1, H5N2, H7N1 and H7N2 subtypes were purified from dk/VP06. Since plaque purification could not be carried out directly from clinical material, the five subtypes and eight different genotypes may not have been present in this constellation in the clinical samples. Some of the sub- and genotypes detected following plaque purification could have been generated during virus isolation in ECE and/or plaque-passaging. Surprisingly, no replicating H9N2 virus was rescued during plaque purification from dk/VP06 although RITA confirmed the presence of this subtype in this sample as well (Table 1). We speculate that this might have been due to the interference of the different viruses in this sample, and a higher replication efficiency of the numerous H5 and H7 viruses which might have outcompeted the H9 virus in our cloning approach.

In depth molecular analyses revealed that the rescued H5N1 and H5N2 viruses derived their HA H5 segment from clade 2.3.2.1a viruses of the years 2012–2014 (HA, Fig. 2, H5), while the N1 segment along with six internal genes most likely originated from clade 2.2.2/H5N1 (Fig. 2, N1). However, the H5N1 viruses of this clade became apparently extinct from Bangladesh after 2011^14,15^. Continued circulation of genome segments of that clade therefore was unexpected and currently stands as a solitary but important finding. As such, the possibility of undetected circulating clade 2.2.2 HPAI viruses or gene segments thereof in other reassortant subtypes must be considered. Chicken and duck samples (C1/2016 and D1/2016) obtained in the Mymensingh district in late 2016 and treated similarly in our labs proved to contain each a single AIV strain which carried contemporary 2.3.2.1a HA and NA genome segments (Fig. 2, H5).

Even more surprising was the finding of an HPAI H7 virus having an HA gene similar to that of HPAI Pak/H7N3 viruses that had caused outbreaks in Pakistan during 2000–2003^24^, but which had not been detected at that or any time in Bangladesh. Although subtype H7 viruses have been detected in backyard and free ranging ducks in Bangladesh, they were of low pathogenicity and their HA segment was of a Eurasian wild bird lineage unrelated to the HP H7 Pakistan viruses^7,25^ (Fig. 2, H7).

A generic explanation for the detection of genome segments of older HP H5 and HP H7 strains in these samples would be by contamination in the laboratory. However, this can be rejected as a single diagnostic laboratory in Bangladesh (FVS-BAU) undertook all sample manipulations including virus isolation and this laboratory did never have access to replication-competent HP Pak/H7 and Bangladeshi HP H5N1 viruses of clade 2.2.2. Likewise, none of these viruses were available in the laboratory at FLI where subsequent RITA assays, virus amplification and plaque purification experiments were carried out. Unfortunately, original tissues of these cases were no longer retrievable for confirmation. Thus, the true sources of the HP H7 and HP H5 2.2.2 viruses remain elusive.

HA and NA segments of purified H9N2 clones were similar to circulating contemporary G1/H9N2 viruses although their internal genes originated from clade 2.2.2/H5N1. Previously, it was also reported that Bangladeshi H9N2 viruses shared internal genes with HP H5N1 (PB1, clade 2.3.2.1a) and with the HP Pak/H7N3^11–13^. These findings at least indicate independent of our findings that there must have been opportunities of reassortment between HP H5N1 and the Pakistani H7 viruses although it remained unclear where and when such events took place. In turn, our findings could provide a very comprehensible explanation for those observations. Also in Cambodia, extensive co-circulation of and reassortment among H5, H7 and H9 AIV in poultry has recently been pointed out^26^.

The potential of sustained replication in ECE of G1/H9N2 viruses even in competition with HPAI H5 and H7 viruses was demonstrated during a limited number of passages in co-infected ECE. Although no prognosis for the next 10 or 50 passages can be made, it seems conceivable that sets of different AIV sub- and genotypes may be co-transmitted across at least several passages also *in vivo*. Yet, it is not possible to extrapolate these results to the field where additional transmission bottlenecks may exist. Stable replication in chickens of an LPAI H7N7 virus even in presence of its HP mutant descendant virus has recently been reported^27^.

From a diagnostic perspective, the presence of minor viral populations within extended mixtures as described here would only be detected by using highly sensitive and multiple subtype-discerning diagnostic tools such as RITA. In contrast, more global diagnostic approaches including direct Sanger sequencing based on universal “Hoffmann” primers and strain-specific sequencing primers might yield incomplete and even false conclusions depending on the specificity of the primers. This was initially the case here, when different reassorted H9N2 viruses were diagnosed on basis of full-length genome Sanger sequencing. In addition, also high throughput sequencing approaches might give more insights for those samples in the future.

Presence of HPAIV phenotypes in G1/H9N2 mixtures would be expected to influence pathogenesis and clinical picture. This was evident also here with the detection of signs of systemic infection and lesions in multiple organs (Fig. 1), but comparatively low cumulative mortality in affected flocks. Viral population size within those mixture and, hence, clinical sequelae, would vary according to the specific immune status of the hosts. In the reported cases, the affected layer chickens probably had received H5-specific vaccination. Recently, chickens and ducks sold in live bird market in Bangladesh were found positive for HPAIV H5N1 but appeared apparently healthy when sampled although the vaccination status of those birds was unknown^28^. Potential masking of HPAIV related clinical signs in vaccinated or LPAIV co-infected poultry should be considered when analyzing spread of HPAIV along trading networks.

The much unexpected findings of mixtures of reassorted HP H5N1 and G1-like H9N2 viruses which carry genome segments of older gs/GD clades and the detection of HP H7 HA segments previously not reported from Bangladesh, calls for confirmation of these results by targeted surveillance in the area of origin of the investigated samples. Virus strains or reassorted gene segments thereof believed to be extinct may yet still reside in small regional pockets or populations. In general, our findings emphasize the need of a broader and deeper analysis of (AI) viral populations in clinical samples and corresponding viral isolates originating from AI outbreaks. While next-generation sequencing techniques provide ultimate tools for this approach, available multiplexed or arrayed RT-qPCR applications allow for a rapid screening at more sensible costs and of higher sample numbers.

## Methods

### Sample origin, pathology and virus propagation

Necropsy, histopathology and virological investigations as previously described^29^ were carried out upon arrival of poultry carcasses at the Department of Pathology, Faculty of Veterinary Science, Bangladesh Agricultural University (FVS-BAU). In addition, presence of AIV and Newcastle Disease virus (NDV) RNA, respectively, in lung and trachea tissue was investigated by conventional RT-PCR targeting fragments of the matrix (M) gene of both AIV and NDV^30,31^. Full-length genome sequences were established using the Hoffmann primers^32^ as detailed below. Tissue homogenates (lung and trachea, other tissues or sera were not made available) were inoculated into 10-day old embryonated chicken eggs (ECE) following standard procedures^33^. Allantoic fluid of this egg passage was sent to the Friedrich-Loeffler-Institute (FLI) for further investigations. At FLI, passaged samples were HA- and NA-subtyped using the Riems influenza A typing assay (RITA)^34^.

### Plaque purification in Madin-Darby canine kidney (MDCK-II) cell cultures

Based on the RITA results three rounds of plaque purification were conducted for two selected egg-grown virus isolates (ck/VP02 and dk/VP06) to obtain pure virus clones. Briefly, 100 µL of 10-fold serial dilutions of each isolate were incubated on confluent MDCK-II (ATCC® CRL-2936™) cell monolayers in six well plates for 1 h at 37 °C. Cells were carefully washed twice with phosphate buffered saline (PBS) and then overlaid with modified Eagle’s medium containing 1.8% agar, 3.5% bovine serum albumin and 1 μg/mL N-tosyl L-phenylalanine chloromethyl-ketone (TPCK)-treated trypsin (Sigma T1426). After three days of incubation at 37 °C, 5% CO_2_, viral plaque formation was confirmed and individual clones were selected under the light microscope. Virus clones selected were not amplified between three plaque passages. Selected virus clones of the third plaque passage were finally amplified in ECE for production of virus stocks. Plaque clone harvest at different passages and their selection for further analyses are shown in Supplementary Data; Table S4. Plaque morphology of these stocks was recorded after 10% formalin fixation and 1% crystal violet staining.

### Full-genome sequencing and phylogenetic analyses

At FVS-BAU, universal influenza primer sets^32^ for each genome segment were used for full-length amplification of RNA extracted from clinical samples VP02-VP06. Specific primer sets were used for cycle sequencing. At FLI, Sanger sequencing of full-length HA and NA was also performed for all plaque-purified viruses. In addition, whole-genome sequences were established for the H7N1 and H7N2 plaque-purified clones. For the H5 and H9 plaque-purified clones no full-length sequences of internal genes were generated due to the excessive amount of sequencing work. Instead, as shown below, a SYBR-Green-based RT-qPCR technique was used to define their backbone provenance (either H5 or H9). RT-PCR, amplificate purification and cycle sequencing were carried out as described previously^35^. Sequences were assembled and edited using the Geneious software, version 10.2.3^36^. Alignment and identity matrix analyses were performed using MAFFT^37^. Sequences generated in this study were deposited in NCBI GenBank and in the Global Initiative on Sharing All Influenza Data (GISAID) database (accession numbers, Supplementary Data; Table S5). Sequences of other AIV required for the analyses were extracted from public database. Maximum likelihood phylogenetic analysis of manually edited alignments of full-length open reading frames was carried out using the IQ Tree software, version 1.6.7^38,39^. Trees were viewed and edited using the FigTree v1.4.2 software (http://tree.bio.ed.ac.uk/software/figtree/) and Inkscape 0.92.

### Genotype characterization by segment-specific SYBR-Green-based RT-qPCR assays

For rapid genotyping, segment-specific SYBR-Green RT-qPCR assays were established. Based on full-length genome data of clinical isolates and some of the plaque-purified viruses, two sets of SYBR-Green RT-qPCRs were designed; one specific for internal segments of the H5N1 clade 2.2.2 lineage, the other specific for the G1/H9N2 lineage. Primers were selected using Oligo Calculator, online version 3.26 (Supplementary Data; Table S6)^40^. Adjusting the assays and validation of specificity using the qScript™One-Step SYBR-Green kit (Quanta BioSciences, USA) was carried out with homologous and heterologous gene segments of fully sequenced reference isolates. Melting point analysis of the amplificates was used as an additional specificity control as described^41^.

### Calculation of TCID_50_ and Mean Death Time (MDT)

The infectivity titer of viruses was determined by microtitration in MDCK-II cells as described before^42^. Virus-induced cytopathic effects were analysed after an incubation period of 72 hrs and titers were calculated by an adapted Kärber-Spearman method^43^. For mean death time (MDT) measurements of plaque-purified viruses, five ECE per virus were inoculated each with a fixed concentration of 10^3^ TCID_50_ and candled twice daily.

### *In ovo* virus competition assay

Co-inoculation into 14-days old embryonated chicken egg (ECE) at 10^3^ TCID_50_ per inoculated virus was used to compare viral fitness of the different sub- and genotypes in 16 combinations each of two viruses obtained during plaque purification. In these combinations all five subtypes and eight genotypes were included. In an additional co-inoculation experiment, three viruses were combined, HP H5N1, HP H7N2 and H9N2. Three consecutive blind passages each of 48 hours in ECE were carried out using 200 µL of allantoic fluid diluted at 10^−4^ as inoculum for onward passages. RNA was extracted at passages 0 (P0) and 3 (P3) for sub- and genotyping using RT-qPCRs as detailed above selecting PCRs that are specific for those segments that distinguished between the two co-inoculated virus clones. Cq-values were determined as a relative measure of the proportion of the distinct segments in the mixture.

### Ethical statement

We state that none of the work carried out in this study required approval from ethics committees as neither human samples nor animal experiments were employed. All experiments and methods were performed with approval and in accordance with the respective relevant legal guidelines and regulations of Bangladesh Agricultural University, Bangladesh, and the Friedrich-Loeffler-Institut, Germany.

## Supplementary information

Supplementary Data

## Data Availability

The accession numbers of all the sequences generated and deposited in NCBI GenBank and in the Global Initiative on Sharing All Influenza Data (GISAID) database, all designed primer used in this study and other datasets analyzed during this study are provided in the Supplementary Data of this article.
