# Supplementary material for: Co-subsistence of avian influenza virus subtypes of low and high pathogenicity in Bangladesh: Challenges for diagnosis, risk assessment and control
Source: Sci Rep. 2019 Jun 5;9:8306. doi: 10.1038/s41598-019-44220-4 (PMC6549172; doi:10.1038/s41598-019-44220-4)
Supplement: Supplementary file 1 — Supplementary Data [file 41598_2019_44220_MOESM1_ESM.docx]

**Supplementary Files**

**Co-subsistence of avian influenza virus subtypes of low and high pathogenicity in Bangladesh: Challenges for diagnosis, risk assessment and control**

Rokshana Parvin, Jahan Ara Begum, Emadadul Haque Chowdhury, Mohammed Rafiqul Islam, Martin Beer and Timm Harder

| **Virus** | **Receptor binding Site (RBS)**  **190 helix** | **Left RBS**  **pocket** | **Right RBS**  **pocket** | **Potential glycosylation site** | | | | | | | | | **Cleavage site** |
| --- | --- | --- | --- | --- | --- | --- | --- | --- | --- | --- | --- | --- | --- |
| H9-HA (H9 numbering) | 198-206 | 232-237 | 146--150 | 29 | 105 | 141 | 206 | 218 | 298 | 305 | 492 | 551 | 333-341 |
| A/quail/Hong Kong/G1/97 (H9N2) | EQTNLYIRN | NDLQGR | GISRA | + | + | + | + | + | + | + | + | + | PARSSR/GLF |
| A/layer chicken/Bangladesh/VP02/2016 (Mixed) | AQTNLYTRT | NGLIGR | GTSKS | + | + | + | - | - | + | + | + | + | PAKSKR/GLF |
| A/layer chicken/ Bangladesh/VP03/2016 (Mixed) | AQTNLYTRT | NGLIGR | GTSKS | + | + | + | - | - | + | + | + | + | PAKSKR/GLF |
| A/layer chicken/ Bangladesh/VP04/2016 (Mixed) | AQTNLYTRT | NGLIGR | GTSKS | + | + | + | - | - | + | + | + | + | PAKSKR/GLF |
| A/layer chicken Bangladesh/VP05/2016 (Mixed) | TQTNLYTRT | NGLIGR | GTSKS | + | + | + | - | - | + | + | + | + | PAKSKR/GLF |
| A/duck/ Bangladesh/VP06/2016 (Mixed) | AQTNLXTRT | NGQIGR | GTSKS | + | + | + | - | - | + | + | + | + | PAKSKR/GLF |
| A/chicken/Bangladesh/VP02-Plaque purified H9A/2016 (H9N2) | AQTNLYTRT | NGLIGR | GTSKS | + | + | + | - | - | + | + | + | + | PAKSKR/GLF |
| H5-HA (H5 numbering) | 202-210 | 233-241 | 146-150 | 27 | 39 | 156 | 181 | 302 | 500 | 559 |  |  | 337-349 |
| A/Goose/Guangdong/1/96 (H5N1) | EQTKLYQNP | PKVNGQSGR | GVSSA | + | + | - | + | + | + | + |  |  | PQRERRRKKRGLF |
| A/chicken/Bangladesh/FDIL(G)514/2007 (H5N1) c2.2.2 | EQTRLYQNP | SKVNGQSGR | GVSSA | + | + | - | + | + | + | + |  |  | PQGERRRKKRGLF |
| A/chicken/Bangladesh/12VIR-7140-14/2012 (H5N1) c2.3.2.1a | EQTRLYQNP | PKINGQSGR | GVSAA | + | + | + | + | + | + | + |  |  | PQRERRR-KRGLF |
| A/chicken/Bangladesh/VP02-Plaque purified H5A/2016 (H5N1) | EQTRLYQNP | SKINGQSGR | GVSAA | + | + | + | + | + | + | + |  |  | PQRERRR-KRGLF |
| A/chicken/Bangladesh/VP02-Plaque purified H5C/2016 (H5N2) | EQTRLYQNP | SKINGQSGR | GVSAA | + | + | + | + | + | + | + |  |  | PQRERRR-KRGLF |
| A/duck/Bangladesh/VP06-Plaque purified H5B/2016 (H5N1) | EQTRLYQNP | SKINGQSGR | GVSAA | + | + | + | + | + | + | + |  |  | PQRERRR-KRGLF |
| A/duck/Bangladesh/VP06-Plaque purified H5D/2016 (H5N2) | EQTRLYQNP | SKINGQSGR | GVSAA | + | + | + | + | + | + | + |  |  | PQRERRR-KRGLF |
| A/duck/Bangladesh/VP06-Plaque purified H5E/2016 (H5N2) | EQTRLYQNP | SKINRQSGR | GVSAA | + | + | + | + | + | + | + |  |  | PQRERRR-KRGLF |
| H7-HA (H7 numbering) | 199-207 | 230-238 |  | 30 | 46 | 141 | 423 | 495 |  |  |  |  | 333-344 |
| A/chicken/Karachi/NARC-100/2004 (H7N3) | EQTRLYGSG | PQVNGQSGR |  | + | + | - | + | + |  |  |  |  | PETPKRRKRGLF |
| A/duck/Bangladesh/VP06-Plaque purified H7A/2016 (H7N1) | EQTRLYGSG | PQVNGQSGR |  | - | + | + | + | + |  |  |  |  | PETPKRRKRGLF |
| A/duck/Bangladesh/VP06-Plaque purified H7B/2016 (H7N2) | EQTRLYGSG | PQVNGQSGR |  | - | + | + | + | + |  |  |  |  | PETPKRRKRGLF |

**Table S1** Receptor binding (RBS), potential glycosylation and cleavage sites profiles of the HA of avian influenza viruses characterized before and after plaque purification isolated from poultry in Bangladesh in 201

**Table S2.** Molecular marker analysis of PB2, PB1, PA, NP, M and NS genes (H9 numbering) of recent Bangladeshi field isolates.

| Selected strains | M | | | | | NS1 | | | | | | | NP | | PA | | | | | PB1 | | | | PB2 |
| --- | --- | --- | --- | --- | --- | --- | --- | --- | --- | --- | --- | --- | --- | --- | --- | --- | --- | --- | --- | --- | --- | --- | --- | --- |
|  | M1 | M2 | | | |  |  |  |  |  |  |  |  |  |  |  |  |  |  | PB1 | | | PB1-F2 |  |
|  | 15 | 26 | 27 | 31 | 55 | Deletion of aa 80-84 | PDZ motif | 42 | 92 | 103 | 106 | 227 | 136 | 372 | 55 | 337 | 367 | 409 | 550 | 13 | 336 | 375 | 66 | 627 |
| Q/HK/G1/97 | I | L | V | S | F | No | EPEV | S | E | L | I | E | M | E | D | A | M | S | L | P | V | N | N | E |
| Lyr Ck/BD/VP02/16 | I | L | V | S | L | No | KSEV | S | D | F | M | K | I | D | D | T | R | S | I | P | V | N | N | K |
| Lyr Ck /BD/VP03/16 | I | L | V | N | F | No | KSEV | S | D | F | M | K | I | D | D | T | K | S | L | P | V | T | N | K |
| Lyr Ck /BD/VP04/16 | I | L | V | N | F | No | KSEV | S | D | F | M | K | L | E | D | T | R | S | I | P | V | N | N | K |
| Lyr Ck /BD/VP05/16 | I | L | V | N | F | No | KSEV | S | D | F | M | K | L | E | D | T | E | S | I | P | V | T | N | E |
| Dk/BD/VP06/16 | I | L | V | S | L | Yes | ESKV | S | D | F | M | E | L | E | D | T | E | S | L | P | V | N | N | K |

*Abbreviation: Q - quail; HK - Hongkong; Lyr Ck - layer chicken; BD - Bangladesh

**Table S3**. In ovo genome competition assay: Comparison of C_q_ values at passages 1 (P1) and 3 (P3) for M, and subtype-specific HA and NA segments.

| **Co-inoculated strains** | | **M** | | **HA-H5** | | **HA-H9** | | **HA-H7** | | **NA-N1** | | **NA-N2** | |
| --- | --- | --- | --- | --- | --- | --- | --- | --- | --- | --- | --- | --- | --- |
| **Genotype** | **Subtype** | **P0** | **P3** | **P0** | **P3** | **P0** | **P3** | **P0** | **P3** | **P0** | **P3** | **P0** | **P3** |
| H5 A/B | H5N1+H5N1 | 19,54 | 13,4 | 21,33 | 14,3 |  |  |  |  | 20,7 | 15,62 | N/A | N/A |
| H5 C/D | H5N2+H5N2 | 18,98 | 13,75 | 20,23 | 13,97 |  |  |  |  | N/A | N/A | 20,42 | 13,64 |
| H5 C/E | H5N2+H5N2 | 18,52 | 13,67 | 20,04 | 14,16 |  |  |  |  | N/A | N/A | 19,96 | 13,99 |
| H5 D/E | H5N2+H5N2 | 18,22 | 14,19 | 19,79 | 14,26 |  |  |  |  | N/A | N/A | 19,71 | 14,13 |
| H5 A/C | H5N1+H5N2 | 19,11 | 12,9 | 20,52 | 13,48 |  |  |  |  | 21,13 | 19,87 | 21,34 | 13,29 |
| H5 A/D | H5N1+H5N2 | 20,11 | 12,29 | 21,25 | 13,59 |  |  |  |  | 23,77 | N/A | 21,16 | 13,37 |
| H5 A/E | H5N1+H5N2 | 19,49 | 11,79 | 20,55 | 13,23 |  |  |  |  | 23,72 | 21,3 | 20,39 | 13,18 |
| H5 B/C | H5N1+H5N2 | 20,29 | 13,15 | 20,65 | 14,06 |  |  |  |  | 22,86 | 20,36 | 21,39 | 15,2 |
| H5 B/D | H5N1+H5N2 | 20,21 | 12,76 | 21,01 | 13,88 |  |  |  |  | 22,93 | 21,23 | 21,03 | 14,12 |
| H5 B/E | H5N1+H5N2 | 19,84 | 12,52 | 20,38 | 13,72 |  |  |  |  | 22,95 | 23,37 | 20,43 | 16,65 |
| H9A/H5A | H9N2+H5N1 | 15,32 | 15,23 | 23,53 | 13,59 | 16,77 | 19,73 |  |  | 20,46 | N/A | 15,79 | 15,2 |
| H9A/H5B | H9N2+H5N1 | 15,07 | 15,84 | 22,78 | 13,23 | 16,15 | 15,5 |  |  | 26,74 | N/A | 15,63 | 14,69 |
| H9A/H5C | H9N2+H5N2 | 14,95 | 15,75 | 21,31 | 14,06 | 15,99 | 14,46 |  |  | N/A | N/A | 15,34 | 14,02 |
| H9A/H5D | H9N2+H5N2 | 15,24 | 18,68 | 21,77 | 13,88 | 16,46 | 18,4 |  |  | N/A | N/A | 18,06 | 15,73 |
| H9A/H5E | H9N2+H5N2 | 15,01 | 17,94 | 20,96 | 13,72 | 16,14 | 18,21 |  |  | N/A | N/A | 17,01 | 15,56 |
| H5B/H7A/H9A | H5N1+H7N1+H9N2 | 15,79 | 12,46 | 23,44 | 15,13 | 16,48 | 14,61 | 25,74 | 14,83 | 27,22 | 14,68 | 16,11 | 13,38 |

**Table S4**. Course of plaque purification of sub- and genotypes

| **Isolate** | **Subtype** | **Plaque passages** | | | | **Selected** |
| --- | --- | --- | --- | --- | --- | --- |
|  |  | **P1** | **P2** | **P3** | **Total** |  |
| ck/VP02 | H5N1 | 26 | 21 | 17 | 64 | 10 |
|  | H5N2 | 29 | 22 | 18 | 69 | 10 |
|  | H9N2 | 13 | 11 | 5 | 29 | 10 |
| dk/VP06 | H5N1 | 19 | 15 | 10 | 44 | 10 |
|  | H5N2 | 31 | 12 | 13 | 56 | 10 |
|  | H7N1 | 7 | 5 | 5 | 17 | 5 |
|  | H7N2 | 16 | 7 | 5 | 28 | 5 |
|  | H9N2 | 0 | 0 | 0 | 0 | 0 |

**Table S5.** Identity and accession numbers of Bangladeshi AIV field isolates (all eight segments) and plaque-purified clones (HA and NA segments) thereof sequenced in the frame of the current study.

| **No** | **Sample ID** | **Host species** | **Source** | **Gene segment** | **Accession Number** |
| --- | --- | --- | --- | --- | --- |
| 1 | A/Layer chicken/Bangladesh/VP02/ 2016 (Mixed) | Chicken | Farm | PB2 | MG645316 |
|  |  |  |  | PB1 | MG645330 |
|  |  |  |  | PA | MG645378 |
|  |  |  |  | HA | MG599733 |
|  |  |  |  | NP | MG645333 |
|  |  |  |  | NA | MG602521 |
|  |  |  |  | M | MG602526 |
|  |  |  |  | NS | MG645325 |
| 2 | A/Layer chicken/Bangladesh/VP03/ 2016 (Mixed) | Chicken | Farm | PB2 | MG645317 |
|  |  |  |  | PB1 | MG645331 |
|  |  |  |  | PA | MG645379 |
|  |  |  |  | HA | MG599734 |
|  |  |  |  | NP | MG645334 |
|  |  |  |  | NA | MG602522 |
|  |  |  |  | M | MG602527 |
|  |  |  |  | NS | MG645326 |
| 3 | A/Layer chicken/Bangladesh/VP04/ 2016 (Mixed) | Chicken | Farm | PB2 | MG645318 |
|  |  |  |  | PB1 | MH828371 |
|  |  |  |  | PA | MG645380 |
|  |  |  |  | HA | MG599735 |
|  |  |  |  | NP | MH828370 |
|  |  |  |  | NA | MG602523 |
|  |  |  |  | M | MG602528 |
|  |  |  |  | NS | MG645327 |
| 4 | A/Layer chicken/Bangladesh/VP05/ 2016 (Mixed) | Chicken | Farm | PB2 | MG645319 |
|  |  |  |  | PB1 | MH828373 |
|  |  |  |  | PA | MG645381 |
|  |  |  |  | HA | MG599736 |
|  |  |  |  | NP | MH828372 |
|  |  |  |  | NA | MG602524 |
|  |  |  |  | M | MG602529 |
|  |  |  |  | NS | MG645328 |
| 5 | A/Duck/Bangladesh/VP06/ 2016 (Mixed) | Duck | Backyard | PB2 | MG645320 |
|  |  |  |  | PB1 | MH828375 |
|  |  |  |  | PA | MG645382 |
|  |  |  |  | HA | MG599737 |
|  |  |  |  | NP | MH828374 |
|  |  |  |  | NA | MG602525 |
|  |  |  |  | M | MG602530 |
|  |  |  |  | NS | MG645329 |
| 6 | A/layer chicken/Bangladesh/VP02-Plaque H9A/ 2016 (H9N2) | Chicken | Farm | HA & NA | EPI1318137  EPI1318138 |
| 7 | A/layer chicken/Bangladesh/VP02-Plaque H5A/ 2016 (H5N1) | Chicken | Farm | HA & NA | EPI1319687  EPI1319688 |
| 8 | A/layer chicken/Bangladesh/VP02-Plaque H5C/ 2016 (H5N2) | Chicken | Farm | HA & NA | EPI1319689  EPI1319690 |
| 9 | A/duck/Bangladesh/VP06-Plaque H5B/ 2016 (H5N1) | Duck | Backyard | HA & NA | EPI1319691  EPI1319692 |
| 10 | A/duck/Bangladesh/VP06-Plaque H5D/ 2016 (H5N2) | Duck | Backyard | HA & NA | EPI1319693  EPI1319694 |
| 11 | A/duck/Bangladesh/VP06-Plaque H5E/ 2016 (H5N2) | Duck | Backyard | HA & NA | EPI1319695  EPI1319696 |
| 12 | A/duck/Bangladesh/VP06-Plaque H7A/ 2016 (H7N1) | Duck | Backyard | HA & NA  PB2-NS | EPI1319697  EPI1319698  EPI1322948-53 |
| 13 | A/duck/Bangladesh/VP06-Plaque H7B/ 2016 (H7N2) | Duck | Backyard | HA & NA  PB2-NS | EPI1319699  EPI1319700  EPI1322954-59 |
| 14 | A/duck/Bangladesh/AR132-18 D1/2016 (H5N1) | Duck | Backyard | HA & NA | EPI1322960  EPI1322961 |
| 15 | A/chicken/Bangladesh/AR134-18 C1/2016 (H5N1) | Chicken | Farm | HA & NA | EPI1322962  EPI1322963 |

^*^Mixed – Although subtype H9N2 was determined by direct Sanger sequencing, later in-depth examination by RITA revealed presence of further subtype in the same samples.

**Table S6.** Primers used in SYBR-green RT-qPCR assays for differentiation of internal segment origin from H9N2 (G1 lineage) or H5N1 (clade 2.2.2) viruses circulating in poultry in Bangladesh.

| **Primer** | **Sequence** | **Segment** | **Position^1^** | **Amplicon size** | **Accession no.** |
| --- | --- | --- | --- | --- | --- |
|  |  |  |  |  |  |
| H9-PB2-F | CCGAAGCATTGTYAGAAG G | PB2 | 792-810 | 68 | MG042198 |
| H9-PB2-R | CTATGGCACATTTCCAAGAGT |  | 860-840 |  |  |
|  |  |  |  |  |  |
| H9-PB1-F | GCGAGATTGGGAAAAGGT | PB1 | 1045-1062 | 158 | MG042359 |
| H9-PB1-R | GGCTGTACCATCTATTAGG |  | 1203-1185 |  |  |
|  |  |  |  |  |  |
| H9-PA-F | TGC TTC ATG TAT TCA GAC TTC | PA | 133-155 | 133 | MG042467 |
| H9-PA-R | GTC CAG GCC AAT GTT CGA |  | 266-249 |  |  |
|  |  |  |  |  |  |
| H9-NP-F | CAG CCC ACT TTC TCA GTG | NP | 1258-1275 | 131 | MG042083 |
| H9-NP-R | TCT GGC ACT TTC CAT CAT C |  | 1389-1371 |  |  |
|  |  |  |  |  |  |
| H9-M-F | GAT GGG AAC AGT GAA CAC A | M | 415-433 | 116 | MG042338 |
| H9-M-R | ATT AGC GGA TTG GTA GTA GC |  | 531-512 |  |  |
|  |  |  |  |  |  |
| H9-NS-F | GCT CCA CGC TAC CTG AG | NS | 270-286 | 227 | MG042238 |
| H9-NS-R | TGA GAT TTC TCC CAC AAT TGA |  | 497-477 |  |  |
|  |  |  |  |  |  |
| H5-PB2-F | TAC CAA TGG ATC ATC AGA AAC | PB2 | 1648-1668 | 216 | MG645320 |
| H5-PB2-R | CAG TAT CAA ATG TCC CCA G |  | 1864-1846 |  |  |
|  |  |  |  |  |  |
| H5-PB1-F | CGAA ACA CTA GCG AGG AGT | PB1 | 765-783 | 113 | MH828375 |
| H5-PB1-R | GAG TTA GTC ATC ATY TTC CTC |  | 878-858 |  |  |
|  |  |  |  |  |  |
| H5-PA-F | AGA AAA GGA CAT GAC CAA AGA A | PA | 1833-1854 | 187 | MG645382 |
| H5-PA-R | GCA ATT TTC TTG ATT CAG CTG AA |  | 2020-1998 |  |  |
|  |  |  |  |  |  |
| H5-NP-F | CTG GTC TCA CCC ACC TG | NP | 437-453 | 171 | MH828374 |
| H5-NP-R | GTC CCT ACC CCC TTC AC |  | 608-592 |  |  |
|  |  |  |  |  |  |
| H5-M-F | GGC AAC TAT CAC CAA CCC A | M | 534-552 | 188 | MG602530 |
| H5-M-R | CCA GCA CTA GAA TTA GGA TGA |  | 722-702 |  |  |
|  |  |  |  |  |  |
| H5-NS-F | GGC TTC ACG CTA CCT AAC | NS | 281-298 | 227 | MG645329 |
| H5-NS-R | GAG ATT TCT CCC ACA ATT GC |  | 508-489 |  |  |

^1^Refers to numbering used in the segment as identified under “Accession”
